# An Online Tea Fixation State Monitoring Algorithm Based on Image Energy Attention Mechanism and Supervised Clustering (IEAMSC)

**DOI:** 10.3390/s20154312

**Published:** 2020-08-02

**Authors:** Zhiyong Yu, Jin Wang, Tao Zheng, Guodong Lu

**Affiliations:** State Key Laboratory of Fluid Power and Mechatronic Systems, Zhejiang University, Hangzhou 310027, China; k1062@zju.edu.cn (Z.Y.); zhengtao2955@zju.edu.cn (T.Z.); lugd@zju.edu.cn (G.L.)

**Keywords:** supervised clustering, state monitoring, adaptive filtering, attention mechanism, supervised learning

## Abstract

This study aimed at the shortcomings of existing fixation algorithms that are image-based only, and an effective tea fixation state monitoring algorithm was proposed. An adaptive filtering algorithm was used to automatically filter the ineffective information. Using the energy extractor, the complete energy information of each fixation image was extracted. The image energy attention mechanism was used to identify the prominent features, and based on these, the energy data was mapped to generate the data points as the training data. The cluster idea was adopted, and the training data feed the features trainer. The trend center data of the tea processing energy clustering was generated from different color channels. The corresponding decision function was designed which is based on the distance of the cluster center. The fixation degree of each monitoring image set was measured by the decision function. The Euclidean distance of the energy clustering center of the three channels with the same fixation time progressively approached. The triangle formed by these three points had a trend of gradually shrinking, which was first discovered by us. The detection results showed high accuracy compared with the common classification algorithms. It indicates that the algorithm proposed has positive guiding and reference significance.

## 1. Introduction

Tea is the second most consumed beverage in the world [1,2]. It contains antioxidant compounds of polyphenols that helps the body fight harmful free radicals. It is also believed that harmful free radical can lead to cancer and heart disease. Frequent consumption of tea has been proved to lower the incidence of chronic pathologies, like cancer, cardiovascular, and other diseases [3,4,5]. The catechins (a type of natural phenol and antioxidant) in tea are a powerful sterilizing agent, which kills germs and bacteria. Tea prevents food poisoning by fighting against stomach diseases caused by harmful bacteria. Various steps are involved in the tea processing. In detail, one of the main processes is the fixation that is performed to prevent the oxidation at a certain level in order to produce different flavors in the finished tea. This directly affects the quality of the tea. When the moisture content of the fresh tea reaches 68–70%, the fixation process can be established. In the tea automatic production line, the existing way of fixation mainly includes roller [6], microwave [7], and hot air fixation [8]. For the same form of tea, the fixation equipment usually adopts a fixed temperature and time. In this way, the final fixation state of the tea is susceptible, which influences the quantity, temperature, environmental parameters, and water content. In order to improve the processing quality of the tea, online fixation status monitoring is especially important. However, the existing methods mainly depend on experienced workers to judge the status of the tea fixation, which is an urgent problem to be solved for the fully automated production line. However, there are few studies on the monitoring of tea fixation status, and most of the studies on the tea mainly focus on the analysis of finished tea and tea medicinal properties.

Currently, the major procedures used in the tea research includes: spectral analysis [9,10], electronic nose aroma analysis [11,12,13], chemical composition analysis [14,15], neural network analysis [16,17], and image-based texture analysis [18,19], etc.

The partial-least-squares regression (PLSR) approach and the ratio of performance deviation (RPD) were used to analysis and estimate the tea spectral models [20]. It can accurately predict the moisture content, the arubigin components of TRSI and TRSII, and total polyphenol contents. One study [21] used visible and near-infrared (Vis-NIR) spectroscopy to rapidly measure the six main types of lipid-soluble pigments in green tea. The results show that Vis-NIR spectroscopy is a functional tool for the rapid determination of lipid-soluble pigments. An improved weighted partial least squares (PLS) algorithm combined with near-infrared spectroscopy (NIR) was used by another scholar [22] to construct components calibration model. This study demonstrated that the NIR spectroscopy technology could be applied to Chinese black tea(pu-erh) tea quality assessment. Another study [23] used an electronic nose to evaluate the aromas of the tea samples stored in different years, and the combination of principal component analysis (PCA) and linear discriminant analysis (LDA) was used to analyze the different samples. This study proves that electronic nose is effective in detecting tea aroma components. Based on electronic nose aroma analysis, a herbal tea discrimination method was investigated [24]. In this study, it is shown that high measurement temperature leads to better herbal tea discrimination. The proper fixation is the most important assumption to ensure the content of effective chemical components and tea grade. Frequent consumption of tea benefits consumer-health-promoting effects, since it contains neuroactive compounds. When tea reaches the processing stages, it affects the level of these compounds. Scholars have determined that the chemical compositions of tea changes during tea processing [25]. This study provides a comprehensive means to understand the chemical changes during the tea processing, and it is significant to the improvement of both flavor and quality control. The reversed-phase, high-performance liquid chromatographic connected to ultraviolet detection (RP-HPLC/UV) method was developed for 15 phenolic compounds [26]. This method is simple and reliable, and it is suited for the analysis of phenolic compounds. The fermentation process is one of the factors that directly determines the product quality of black tea. The application of machine learning algorithms in quality assurance of the fermentation process of black tea is based on electrical properties [27]. To control color variation during fermentation, computer vision is used to detect color change in black tea in RGB (Red, Green, and Blue), HSV(Hue, Saturation, and Value), or CIE (Commission International Eclairage) LAB color systems [28]. This method is applied to tea fermentation in a stable environment. However, the robustness and adaptability of this algorithm are not very high, and it is susceptible to noise and is not suitable for the fixation process and the dynamic changes therein.

In review, it is challenging to accurately monitor the fixation state of the spectral analysis for non-metallic elements. The modeling, testing, and equipment involved are expensive. The accuracy of the monitoring results is exposed to external factors, so that spectral analysis is not suitable for online monitoring. When electronic nose aroma analysis is accepted, there is no direct relationship between the fixation state and the aroma data. The monitoring results are immensely influenced by volume size and airflow velocity, which are more suitable for offline grade analysis of completed tea. The chemical composition analysis can accurately calculate the content of specific substances, but the analysis process is time-consuming; thus, it is not suitable for the online status monitoring of tea fixation. For deep learning analysis, the type of training samples has a great impact. In order to make the results more accurate, a large number of samples with different fixation times need to be collected. For the circumstances that are not collected in the monitoring, the randomness is massive, and it is inaccurate; hence, it is not suitable for online monitoring. Texture analysis is commonly used in the image feature analysis method and mainly depends on gray level co-occurrence matrix [29,30]. It is an important step in the solving process, and it is used to solve the image co-occurrence matrix, whose calculation amount is h2 × rows × columns, where h is the number of grayscale. Corresponding to the fixation image in this paper, the size and h are 1920 × 1080 and 256, respectively. The calculation amount of 1.36 × 1012 cannot meet the time requirement of online monitoring. If the grayscale is decreased, the accuracy of the result will be significantly reduced.

One of the main aspects is that the quality of the tea fixation is always connected with the color of the tea leaves throughout the fixation process. When the tea meets the requirements, the human eye can accurately judge the color. There are few studies in the monitoring of online fixation state based on tea image. The common tea research methods are not suitable for the image-based monitoring of online fixation state. In this paper, an online tea fixation state monitoring algorithm based on the image energy attention mechanism of supervised iterative clustering was proposed for the first time. Using image information monitoring can significantly reduce costs compared with the spectrum, and it can be more quickly and conveniently integrated into the automated production line. The image information can be collected continuously, and the small amount of calculations can meet the monitoring needs of the online tea fixation state. Only the images of standard time are needed as training samples, which greatly reduces the need for the training samples. This makes the training and the deployment time faster compared to deep learning. For online fixation status monitoring, a single test sample is composed of multiple fixation pictures, which can reduce the interference of randomness and noise so that the accuracy of the online fixation status monitoring is guaranteed.

This paper mainly includes two different parts. The first part introduces of algorithm principles. The method of each step and its corresponding principles are phased in detail. The second part discusses the experiment, which shows the results of each key step and a comparison with the results of the current mainstream methods of image classification under the same conditions.

## 2. Algorithm Principle

As discusses earlier, fixation is one of the key steps in tea processing. Currently, the existing tea fixation machine mainly controls the temperature and time in a certain range to ensure the quality of the tea fixation. In general, the maximum activity temperature of polyphenol oxidase is 52 °C, and the critical passivation temperature is 82 °C. Due to the factors like instability of quantity, difference in initial moisture content in fresh leaves, and delay in heating, different types and seasons of tea often require different fixation temperatures and time. It is not an ideal method to guarantee the quality of tea by ensuring the quantitative temperature and time. For an automated production line, online tea fixation state automatic monitoring is necessary, rather than relying on the judgment of experienced workers to judge.

The algorithm proposed in this paper mainly includes the following three different steps: acquisition of training data, training, and state monitoring. The acquisition of the training data is getting the required training data from the original image. The training is getting the energy change trend and the decision function, and the state monitoring is realizing the constant online fixation state judgment.

**Figure 1:**
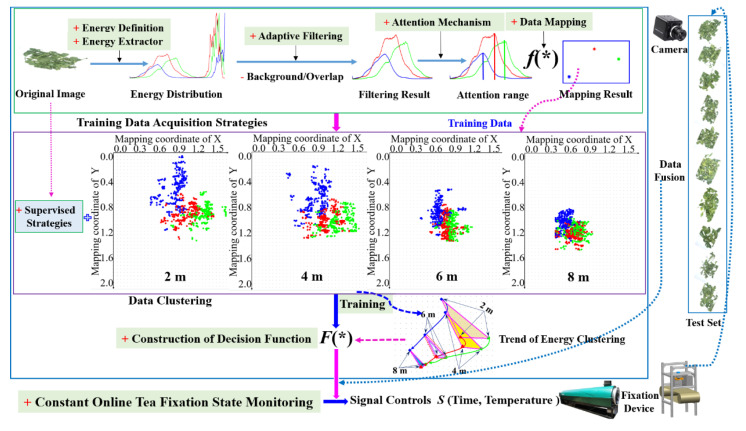
Proposed algorithm detailed steps.

The detailed steps of the proposed algorithm are shown in Figure 1. The proposed algorithm (image energy attention mechanism and supervised clustering (IEAMSC)) mainly includes the follow six steps:

(1) **Energy definition and design of energy extractor**. We define energy as the gray value corresponding to the pixel points in different color channels of an image. If the single channel image is white, the corresponding overall energy is high. Energy extraction is a way to obtain and quantify the original data of fixation images. It is the necessary prerequisite for the state analysis of the tea leaves. The designed energy extractor can extract useful tea information by traversing all color channels in the entire image and count the number of pixels corresponding to the gray value.

(2) **Design of adaptive filter**: The collected tea images have ineffective backgrounds and overlap in the shadow areas, which are not a valuable data for the analysis of tea features, and this obstructs the accuracy of the results. For a single-channel fixation image, we can get two peaks and two troughs in the energy distribution image. From left to right, the first peak position corresponds to the tea leaves information, and the second peak position corresponds to the background information. The first trough is the transition zone between overlapping areas and the tea information, and the second trough is the transition zone between background and tea information. The gray value corresponding to these two points is the two threshold points that we need. The positions of the two points corresponding to each one images are different, so an adaptive filter was designed.

(3) **Attention mechanism of image energy**. The data dimension obtained by filtering is very high, which is difficult to analyze the tea features. In order to reduce the dimension of the data and reflect the main features of the data after dimension reduction, an attentional mechanism of image energy is designed. In the principle of our attention mechanism, the primary focus is the more prominent color feature of an image. After energy extraction, the gray value corresponding to the maximum number of pixels in each channel reflects on the data. 

(4) **Data mapping**. The data obtained from the energy and attention mechanism cannot directly reflect the identification difference of each image. For the subsequent process and analysis, data mapping is the necessary step. The mapping method is to construct a mapping function f(*) whose corresponding variables are the gray value and the number of pixels statistics in the corresponding gray value. The data points of the three different channels can be obtained through the mapping function, and these three points are used as the training data.

(5) **Training**. By feeding the mapping data obtained from different images at the same fixation time into the classifier, we can obtain the feature clustering center belonging to the corresponding fixation time using the obtained cluster center to construct the decision function.

(6) **Construction of decision function and fixation state monitoring**. We can get the standard clustering center based on the training of the fixation images of standard time points or time periods and then get the state decision function F(*). The decision function is constructed by the distance value between three clustering centers corresponding to all training samples at the standard fixation time. The clustering center data of the monitored samples can be substituted into the decision function to accurately judge whether the fixation state is normal or not. 

### 2.1. Energy Extraction and Filter Design

**Figure 2:**
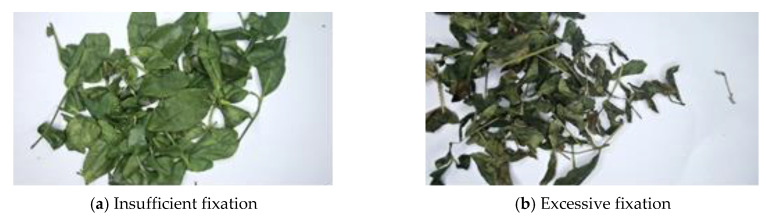
Regional differences in energy concentration of tea fixation image.

The extraction of energy is based on the difference in the overall color features of the image based on the different degrees of tea fixation, which is clearly different from the texture features. The texture relies on the similarity of the adjacent gray level to construct the evaluation matrix. The energy of the tea image with insufficient fixation is mainly concentrated in the green part, while the excessive fixation energy is mainly concentrated in the red, green, and blue parts, as shown in Figure 2.

#### 2.1.1. Definition of Energy 

Energy can be defined as the number of photoelectrons. The greater the number of photoelectrons, the stronger the energy. When the energy projected on the tea leaves is equal (having the same ambient light), the fewer the number of photons absorbed by the tea leaves, and the more the number of photons reflected into the environment. The photons reflected by the tea leaves are captured in the lens. The stronger the energy captured on the photosensitive chip of the camera lens, the brighter the entire image, as shown in the Figure 2a. Conversely, when the tea is over fixation, the fewer photons are captured on the camera chip and the entire image becomes darker, as shown in Figure 2b. 

**Figure 3:**
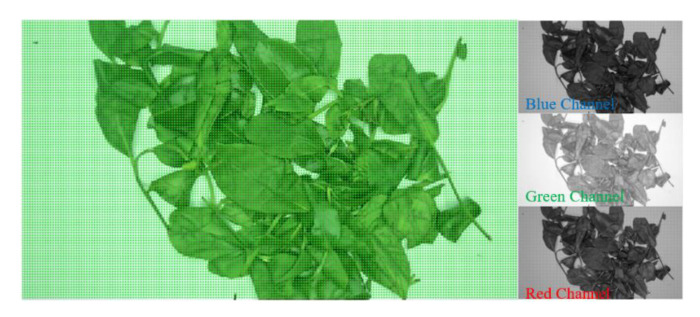
Interpretation of the lightness and darkness of the fixation image.

For the gray scale image, the declaration is bright or dark. Similarly, for the color image, it will show the differences in energy intensity in different color channels. The mathematical expression of light and dark in an image can be assumed as the average percent of the gray value which is the sum of the R, G, and B the three different channels of all pixels. If the average percent is larger, the corresponding image will be brighter and will have higher energy, as shown in Figure 3. It is evidently undesirable to judge the degree of fixation using only the standard of light and dark. It can be judged if only the degree of fixation is very different, and the accuracy rate is easily affected by noise. 

Fixation state monitoring is directly based on the idea of the mean value. The detailed features will be lost. To be more specific, the information that can reflect the trend and difference of the energy distribution in a fixed interval range of the gray levels is unknown. This may easily lead to misjudgment. In this paper, as an energy extractor, the energy-solving process takes the number of pixels corresponding to each gray level in three channels of 256 gray levels. The mathematical model of defining energy is as follows:

(1) $$E_{R,h\in [0,255]}=N_{R,h\in [0,255]}\to \{[if\;\overset{x=rows-1}{\underset{x=0}{\sum }}\overset{y=cols-1}{\underset{y=0}{\sum }}\left(H_{R}(x,y\right)-h)=0]\to (n=1,\underset{h\in [0,255]}{\sum }n=N_{R,h\in [0,255]})\}$$

(2) $$E_{G,h\in [0,255]}=N_{G,h\in [0,255]}\to \{[if\;\overset{x=rows-1}{\underset{x=0}{\sum }}\overset{y=cols-1}{\underset{y=0}{\sum }}\left(H_{G}(x,y\right)-h)=0]\to (n=1,\underset{h\in [0,255]}{\sum }n=N_{G,h\in [0,255]})\}$$

(3) $$E_{B,h\in [0,255]}=N_{B,h\in [0,255]}\to \{[if\;\overset{x=rows-1}{\underset{x=0}{\sum }}\overset{y=cols-1}{\underset{y=0}{\sum }}\left(H_{B}(x,y\right)-h)=0]\to (n=1,\underset{h\in [0,255]}{\sum }n=N_{B,h\in [0,255]})\}$$

where $E_{R,h\in [0,255]}$, $E_{G,h\in [0,255]}$, and $E_{B,h\in [0,255]}$, respectively, represent the energy of different grayscale of color channel R, G, and B in the same picture; $N_{R,h\in [0,255]}$, $N_{G,h\in [0,255]}$, and $N_{B,h\in [0,255]}$ are different channel energy statistical operators; *n* is a constant—only if the gray value of the traversal position is equal to the corresponding *h*, *n* = 1; *H_R_*(*x,y*), *H_G_*(*x,y*), *and H_B_*(*x,y*), respectively, represent the gray values of R, G, and B color channels corresponding to the loop traversal position; rows is the number of picture rows; and cols is the number of picture columns. In image analysis, the pixel point corresponding to the 0th row and the 0th column is used as the starting position of the analysis, and the matrix or array in the data calculation field also starts from 0. In order to maintain consistency, the x and y in the Equation (1)–(3) we designed also start from 0, which is easy to understand and does not make the elements exceed the image size.

#### 2.1.2. Source of Noise and the Design of an Energy Filter

We know that the image often involves background information, and the tea is the foreground. Therefore, background filtering is necessary to minimize the influence of background energy on the detection results. The background color that we used in the experiment is white, which has a higher energy response. 

**Figure 4:**
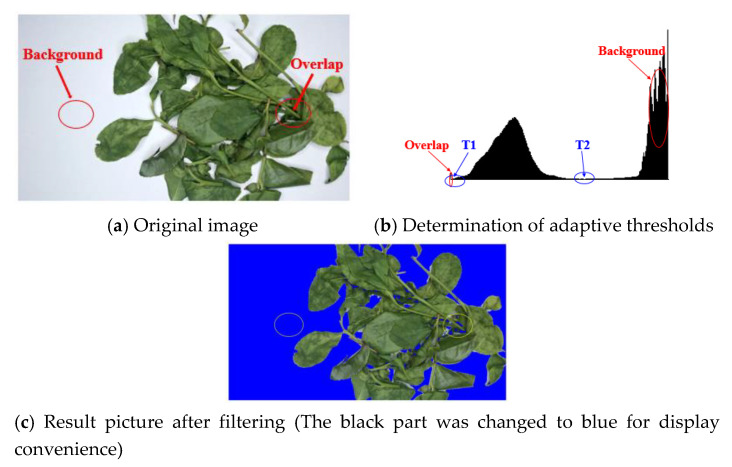
Adaptive dynamic energy filtering.

Generally, the regions where large amount of tea leaves are stacked will form low grayscale areas, as shown in Figure 4a. Hence, these regions are removed as they do not show the reflection of real tea feature. If only the filtering method with fixed threshold value is adopted, some information will vanish in each training picture [31]. Therefore, the adaptive dynamic filtering method was designed.

The principle of adaptive filtering is that the gray levels corresponding to the position of the trough in the energy response spectrum formed by different color channels in each image are contrasting. The background and foreground (tea information) will form two troughs in the entire energy response spectrum. The energy spectrum can be divided into three different parts by taking the x-coordinate of two troughs as the threshold value of the filter (T1 left threshold and T2 right threshold). The useful part in the middle (T1, T2) is the energy features information corresponding to the tea leaves. Therefore, the purpose of adaptive dynamic filtering is achieved. The result is shown in Figure 4, and the mathematical model is as follows:

(4) $$H(x,y)=0.299*H_{R}(x,y)+0.587*H_{G}(x,y)+0.114*H_{B}(x,y)$$

(5) $$T_{1}=h\to \{min(S_{h}\to h),\;[(\overset{x=rows-1}{\underset{x=0}{\sum }}\overset{y=cols-1}{\underset{y=0}{\sum }}H(x,y)(if\;H_{\left(x,y\right)}=h,\;a=1\;else\;a=0\Rightarrow \sum S_{h}=a),\;h\in [0,125])]\}$$

(6) $$T_{2}=h\to \{min(S_{h}\to h),\;[(\overset{x=rows-1}{\underset{x=0}{\sum }}\overset{y=cols-1}{\underset{y=0}{\sum }}H(x,y)(if\;H_{\left(x,y\right)}=h,\;a=1\;else\;a=0\Rightarrow \sum S_{h}=a),\;h\in (125,255])]\}$$

(7) $$\{\begin{array}{c} H_{R,h}(x,y)=0\to \{if\overset{x=rows-1}{\underset{x=0}{\sum }}\overset{y=cols-1}{\underset{y=0}{\sum }}H_{R,h}(x,y)\leq h(h\in [0,T1])||\;H_{R,h}(x,y)\geq h(h\in [T2,255])\}\\ H_{G,h}(x,y)=0\to \{if\;\overset{x=rows-1}{\underset{x=0}{\sum }}\overset{y=cols-1}{\underset{y=0}{\sum }}H_{G,h}(x,y)\leq h(h\in [0,T1])||\;H_{G,h}(x,y)\geq h(h\in [T2,255])\}\\ H_{B,h}(x,y)=0\to \{if\;\overset{x=rows-1}{\underset{x=0}{\sum }}\overset{y=cols-1}{\underset{y=0}{\sum }}H_{B,h}(x,y)\leq h(h\in [0,T1])||\;H_{B,h}(x,y)\geq h(h\in [T2,255])\} \end{array}$$

where *H*(*x, y*) represents the gray value after processing; *S_h_* represents the number of counts corresponding to different grayscale; *a* is the a marker; and *H_R,h_*(*x,y*), *H_G,h_*(*X,Y*), and *H_B,h_*(*x,y*) represent the gray values of the three channels, respectively. The used coefficients in Equation (4) are the weights based on the influence of different color channels on the gray value, and they regarded as the common weight coefficients in the field of image processing to convert an RGB image to a grayscale image.

### 2.2. Design of Training Data 

The training data specified is not the original image data. The image useful feature data is obtained after a series of processing. The dimension of the data is significantly reduced compared with the original image data.

#### 2.2.1. Attention Mechanism

The attention mechanism of the energy concentration is comparable to that of the attention mechanism in machine vision. The target area that needs to be focused on is the so-called focus attention. The main goal is to select the information that is more critical to analysis of the task from the collection of information. 

**Figure 5:**
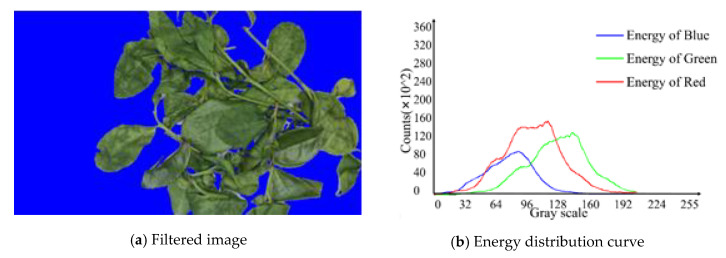
Filtered image data dimension reduction.

The attention mechanism of this algorithm is based on the differences in multiple gray levels in the image. The primary concern is the grayscale range of the energy concentration. In the grayscale range where the energy is concentrated, the different color channels have different features attention ranges. Learning the attention areas in the corresponding images of tea is the various fixation states. Filtered image data dimension reduction is shown in Figure 5.

For an image, the point of our attention mechanism is the average gray levels with the largest number of pixels in the gray level of tea features. In order to decrease the impact of the randomness, we consider *n* gray level from the maximum point to the left and the right. Then, we attain the average to get the energy concentration point. The mathematical model of the attention mechanism of energy concentration is as follows:

(8) $$f_{R}(S_{h})\to \{max(S_{h}\to h),\;[(\overset{x=rows-1}{\underset{x=0}{\sum }}\overset{y=cols-1}{\underset{y=0}{\sum }}H_{R}(x,y)(if\;H_{\left(x,y\right)}=h,\;a=1\;else\;a=0\Rightarrow \sum S_{h}=a),h\in [T1,T2])]$$

(9) $$f_{G}(S_{h})\to \{max(S_{h}\to h),\;[(\overset{x=rows-1}{\underset{x=0}{\sum }}\overset{y=cols-1}{\underset{y=0}{\sum }}H_{G}(x,y)(if\;H_{\left(x,y\right)}=h,\;a=1\;else\;a=0\Rightarrow \sum S_{h}=a),h\in [T1,T2])]$$

(10) $$f_{B}(S_{h})\to \{max(S_{h}\to h),[(\overset{x=rows-1}{\underset{x=0}{\sum }}\overset{y=cols-1}{\underset{y=0}{\sum }}H_{B}(x,y)(if\;H_{\left(x,y\right)}=h,\;a=1\;else\;a=0\Rightarrow \sum S_{h}=a),h\in [T1,T2])]$$

(11) $$\{\begin{array}{c} N_{ave}(R)=\overset{i=c_{\left(R,G,B\right)}+n}{\underset{i=c_{\left(R,G,B\right)}-n}{\sum }}f_{R}(S_{h})/(2n+1)\to (i=c_{\left(R,G,B\right)},\;if\;n=0)\\ N_{ave}(G)=\overset{i=c_{\left(R,G,B\right)}+n}{\underset{i=c_{\left(R,G,B\right)}-n}{\sum }}f_{G}(S_{h})/(2n+1)\to (i=c_{\left(R,G,B\right)},\;if\;n=0)\\ N_{ave}(B)=\overset{i=c_{\left(R,G,B\right)}+n}{\underset{i=c_{\left(R,G,B\right)}-n}{\sum }}f_{B}(S_{h})/(2n+1)\to (i=c_{\left(R,G,B\right)},\;if\;n=0) \end{array}$$

where *N_ave_*(*R*), *N_ave_*(*G*), and *N_ave_*(*B*) are the average count of the range for R, G, and B channel attention concentration. For the tea image with a size of 1920 × 1080, the number of pixels is 2073, 600, and the total number of features of the three channels is 6220, 800. This shows that it is necessary to reduce the dimension of the data. The mechanism of energy concentration is also a method of data dimensionality reduction. The sum is calculated for −n to n, and the boundary is c.

#### 2.2.2. Data Mapping

For different images, the data corresponding to the energy concentration regions are different, and we can map them to a uniform range. The unified scope can more clearly reflect the differences of each image, and this is similar to the normalization process in deep learning. The features remap realizes the transformation and the dimensionality reduction of data because the energy distribution curve data is still high dimensional data. The data mapping function is as follows:

(12) {f(R)=H-int(Nave(R)*(sS)*max(R)/max(R))f(G)=H-int(Nave(G)*(sS)*max(G)/max(G))f(B)=H-int(Nave(B)*(sS)*max(B)/max(B))

(13) $$\{\begin{array}{c} max(R)=f_{R}(S_{h})\\ max(G)=f_{G}(S_{h})\\ max(B)=f_{B}(S_{h}) \end{array}$$

(14) $$\{\begin{array}{c} x_{R}=h\to satisfied\;f_{R}(S_{h})\\ y_{R}=f(R) \end{array}$$

(15) $$\{\begin{array}{c} x_{G}=h\to satisfied\;f_{G}(S_{h})\\ y_{G}=f(G) \end{array}$$

(16) $$\{\begin{array}{c} x_{B}=h\to satisfied\;f_{B}(S_{h})\\ y_{B}=f(B) \end{array}$$

where $x_{R,G,B}$ and $y_{R,G,B}$ represent the corresponding horizontal and vertical coordinates, respectively; *H* = 800, *s* = 700, and *S* = 25,000. The purpose of this setup is to make the results displayed convenient intuitive analysis in a two-dimensional plane; the values of *H*, *s*, and *S* can be modified accordingly.

**Figure 6:**
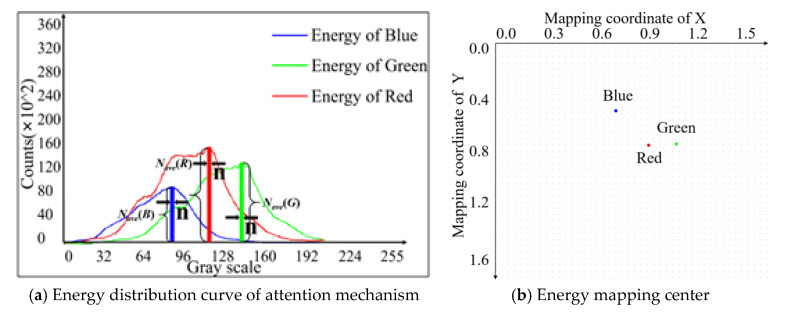
Data mapping.

The values obtained by the mapping function can be used as the x and y values, respectively. We can obtain the coordinate points in each channel energy mapping for a single sample. When *H* = 800, the mapping result drawn on the graph shown in the Figure 6. It is worth observing that the origin of the coordinates in Figure 6b in the upper left corner and the corresponding data are dimensionless.

Therefore, we can get the training data ($x_{R}$*_,_*$y_{R}$), ($x_{G}$*_,_*$y_{G}$), and ($x_{B}$*_,_*$y_{B}$), which is compared with the original image with high dimensional features and which achieves perfect dimension reduction.

### 2.3. Supervised Clustering Training

The algorithm proposed in this paper is based on the supervised energy iterative clustering [32]. It is nothing but all the data in the training contain the fixation label. After processing and mapping each sample data, it is drawn in the same image. We can obtain the image of each batch of fixation mapping following the training. According to the idea of a k-means [33] clustering center, the feature clustering center can be achieved by averaging. In order to make the test results more accurate, we should obtain as many standard images as possible during the training. The sum of squares of errors (*SSE*) is used as the objective function for measuring clustering quality [34]

(17) $$SSE=\overset{k}{\underset{i=0}{\sum }}\underset{x_{i}\in C_{i}}{\sum }dist{\left(c_{i},x\right)}^{2}$$

where *dist* is the standard Euclidean distance (*L*2) between two objects in Euclidean space, and *K* is the number of cluster center. For the above functions, in order to get a better cluster center and minimize the *SSE*, we can minimize the target function to get the *k-th* centroid. That is, find the derivative of *SSE*, make it equal to 0. We get the value of *c_k_*.

(18) $$\frac{\partial }{\partial c_{k}}SSE=\frac{\partial }{\partial c_{k}}\overset{k}{\underset{i=0}{\sum }}\underset{x_{i}\in C_{i}}{\sum }{\left(c_{i}-x\right)}^{2}=\underset{x\in C_{k}}{\sum }2(c_{k}-x_{k})=0$$

(19) $$\underset{x\in C_{k}}{\sum }2(c_{k}-x_{k})=0\Rightarrow m_{k}c_{k}=\underset{x\in C_{k}}{\sum }x_{k}\Rightarrow c_{k}=\frac{1}{m_{k}}\underset{x\in C_{k}}{\sum }x_{k}$$

**Figure 7:**
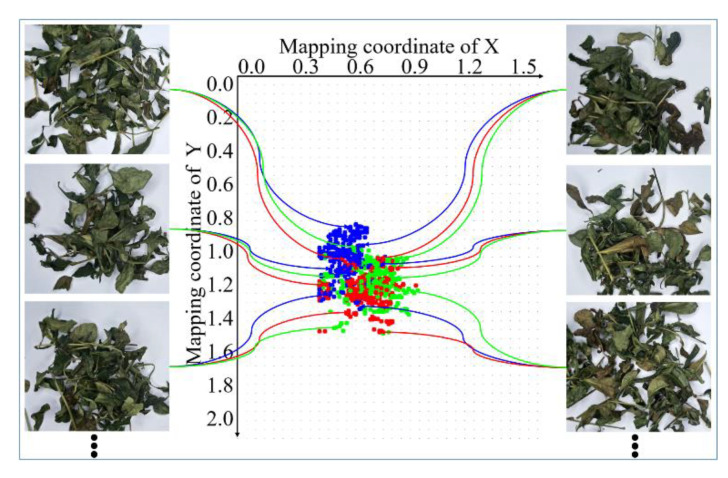
Clustering results of energy at the same fixation time.

It is observed from the above equation that the optimal centroid of the minimum *SSE* is the average value of all the points. Therefore, it is necessary to increase the number of training samples as much as possible. In this way, after the training is complete, the reference standard data distribution will be closer to the ideal distribution. When monitoring, the number of samples in a collection can take 30 or more frames of continuous images within a second. However, the deviation caused by randomness is reduced and the accuracy is improved. The energy clustering results of the same batch samples at the same fixation time are shown in Figure 7. The clustering center of different color channels is the standard point to realize online fixation monitoring.

### 2.4. Realization of Online Tea Fixation State Monitoring

Once the training is completed, the three different standard energy clustering centers are obtained for the standard training set at the same fixation time. Based on the data of each batch of training samples, the three mean energy clustering centers are generated. The online tea fixation state monitoring is generally based on the data of standard center *C*($x_{R}$*_,_*$y_{R}$), *C*($x_{G}$*_,_*$y_{G}$), and *C*($x_{B}$*_,_*$y_{B}$) to judge. It is impossible for the test center to completely correspond to the standard center, and there will be some inaccuracies. We need to set a tolerance range of errors, which can be set according to actual production needs. 

Of course, the training samples can adopt interval (online) and continuous (offline) sampling methods. Because of the proposed algorithm, this paper is based on the standard fixation image as a reference. Only the most suitable fixation samples are needed, which greatly reduces the time of data set preparation and improves the efficiency of practical application. It is also worth observing that the constantly online tea fixation state monitoring one sample is a sequence of images. This helps to ensure that the results are as little affected by single randomness as possible.

**Figure 8:**
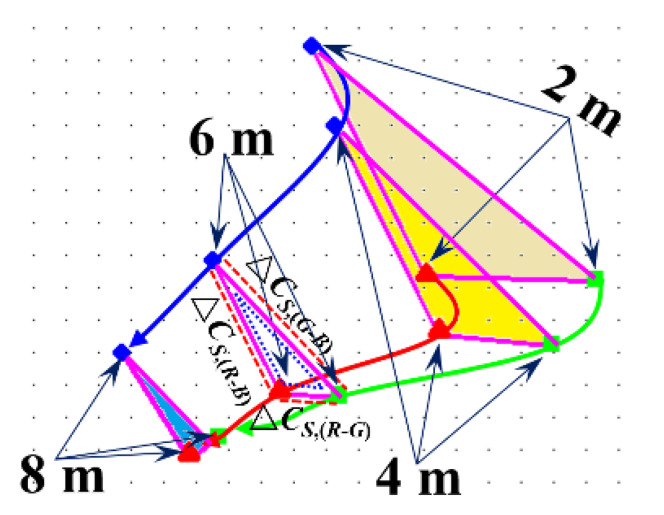
Euclidean distance change and trend of the energy cluster center of the three channels of training samples with the fixation time increased.

Through this study, we establish that, with the increase of fixation time, the Euclidean distance of the energy clustering center of the three channels of the training samples with the same fixation time is gradually approached. The triangle formed by these three points had a trend to gradually shrink in circumference and area, which we first discovered, as shown in Figure 8. Based on this and by comparing the Euclidean distance of the energy clustering center of the training samples and the testing samples, we can realize online tea fixation monitoring.

It is necessary to explain here that the main characteristic of the proposed algorithm is to learn the relative change and trend of the energy and the reasonable range of this tendency rather than learning a fixed numerical feature. 

For training samples, the energy iteration center is

(20) $$\{\begin{array}{c} C_{S,R}=\frac{1}{n}\overset{n=Train}{\underset{n=0}{\sum }}\left(C_{S,R}(n)\right)\\ C_{S,G}=\frac{1}{n}\overset{n=Train}{\underset{n=0}{\sum }}\left(C_{S,G}(n)\right)\\ C_{S,B}=\frac{1}{n}\overset{n=Train}{\underset{n=0}{\sum }}\left(C_{S,B}(n)\right) \end{array}$$

For the energy clustering center of the training sample, the Euclidean distance between different color channel centers is

(21) $$\{\begin{array}{c} \Delta C_{S,(R-G)}=\left|C_{S,R}-C_{S,G}\right|=0.5*{((x_{S,R}-x_{S,G}{)}^{2}+{\left(y_{S,R}-y_{S,G}\right)}^{2})}^{0.5}\\ \Delta C_{S,(R-B)}=\left|C_{S,R}-C_{S,B}\right|=0.5*{((x_{S,R}-x_{S,B}{)}^{2}+{\left(y_{S,R}-y_{S,B}\right)}^{2})}^{0.5}\\ \Delta C_{S,(G-B)}=\left|C_{S,G}-C_{S,B}\right|=0.5*{((x_{S,G}-x_{S,B}{)}^{2}+{\left(y_{S,G}-y_{S,B}\right)}^{2})}^{0.5}\\ sum_{S}=\Delta C_{S,(R-G)}+\Delta C_{S,(R-B)}+\Delta C_{S,(G-B)} \end{array}$$

For testing sample, the energy iteration center is

(22) $$\{\begin{array}{c} C_{M,R}=\frac{1}{n}\overset{n=T\mathrm{est}}{\underset{n=0}{\sum }}\left(C_{M,R}(n)\right)\\ C_{M,G}=\frac{1}{n}\overset{n=T\mathrm{est}}{\underset{n=0}{\sum }}\left(C_{M,G}(n)\right)\\ C_{M,B}=\frac{1}{n}\overset{n=T\mathrm{est}}{\underset{n=0}{\sum }}\left(C_{M,B}(n)\right) \end{array}$$

For the energy clustering center of the testing sample, the Euclidean distance between different color channel centers is

(23) $$\{\begin{array}{c} \Delta C_{M,(R-G)}=\left|C_{M,R}-C_{M,G}\right|=0.5*{((x_{M,R}-x_{M,G}{)}^{2}+{\left(y_{M,R}-y_{M,G}\right)}^{2})}^{0.5}\\ \Delta C_{M,(R-B)}=\left|C_{M,R}-C_{M,B}\right|=0.5*{((x_{M,R}-x_{M,B}{)}^{2}+{\left(y_{M,R}-y_{M,B}\right)}^{2})}^{0.5}\\ \Delta C_{M,(G-B)}=\left|C_{M,G}-C_{M,B}\right|=0.5*{((x_{M,G}-x_{M,B}{)}^{2}+{\left(y_{M,G}-y_{M,B}\right)}^{2})}^{0.5}\\ sum_{M}=\Delta C_{M,(R-G)}+\Delta C_{M,(R-B)}+\Delta C_{M,(G-B)} \end{array}$$

Therefore, the decision function of our online tea fixation state monitoring classifier is

(24) $$F(*)\to if\;\alpha *sum_{S}>\left|sum_{S}-sum_{M}\right|\to \mathrm{monitoring}\;\mathrm{signal}\to \mathrm{normal}$$

(25) $$F(*)\to if\;\alpha *sum_{S}\leq \left|sum_{S}-sum_{M}\right|\to \mathrm{monitoring}\;\mathrm{signal}\to \mathrm{abnormal}$$

where *C_S,R_*(*n*), *C_S,G_*(*n*), and *C_S_*_,*B*_(*n*) are the standard fixation time energy mapping points obtained from a training sample. *C_S,R_*, *C_S_*_,*G*_, and *C_S,B_* are the standard energy clustering center obtained from the all training samples. *C_M_*_,*R*_(*n*), *C_M,G_*(*n*), and *C_M_*_,*B*_(*n*) are the monitoring fixation energy mapping points obtained from the all testing samples of a testing set. *C_M_*_,*R*_, *C_M_*_,*G*_, and *C_M_*_,*B*_ are the monitoring fixation energy clustering center obtained from a testing set. **Δ***C_S_*_,(*R-G*)_, **Δ***C_S_*_,(*R-B*)_, and **Δ***C_S_*_,(*G-B*)_ are the standard fixation energy clustering center distance of three points. **Δ***C_M_*_,(*R-G*)_, **Δ***C_M_*_,(*R-B*)_, and **Δ***C_M_*_,(*G-B*)_ are the monitoring fixation energy clustering center distance of three points. *sum_S_* and *sum_M_* are the total distance. *α* is the weight coefficient. 

## 3. Experimental Verification

The software and hardware environments used in the experiment are as follows. The programming tool is Visual Studio 2017, the machine learning and image processing library is OpenCV-3.4.9, the processor used is the Intel e5–4650, the graphics card is the Nvidia GTX1060, the memory size is ddr3 16 G, and the system is Windows 10 x64 bit. The camera is the fixed focal length camera with a resolution of 5 million pixels and a frame rate of 60 fps. The camera frame rate should be as large as possible to reduce latency.

### 3.1. Collection of Experimental Samples, Fixation Conditions, and Photography Methods

**Figure 9:**
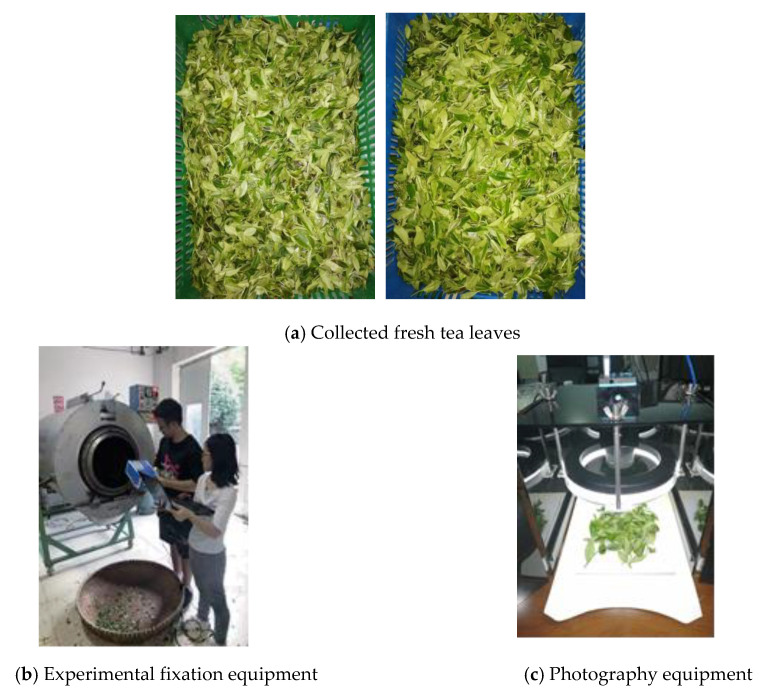
Acquisition of experimental samples.

The fresh tea leaves of Wu Niu Zao and Ying Shuang collected was used to fixation, and the collected fresh tea leaves are shown in Figure 9a. The experimental fixation equipment, 6CWS-90, it is shown in Figure 9b. The fixation conditions in this paper are as follows: temperature 220 °C; rotation speed 25 r/min; and fixation time 2, 4, 6, and 8 min. The photography methods use a focusing lens, as shown in Figure 9c. The main components of the photography equipment include a support structure, a lens, a light, an ambient light-shielding structure, and a camera. The ambient light shielding structure is made from four black polyethylene pipes.

We experiment the samples with fixation time of 2, 4, 6, and 8 min, which includes two categories of green tea—Wu Niu Zao and Ying Shuang. Here, 2800 images were taken as training samples—each time contains 700 images, and the corresponding type of green tea is named Wu Niu Zao. Similarly, 1200 images were taken as test samples, each time contains 300 images, and the corresponding type of green tea is Ying Shuang. A data enhancement technique was used to obtain the test samples from 1200 to 3000 images. 

### 3.2. Verification of the Variation Trend of Image Energy and Energy Clustering Center

**Figure 10:**
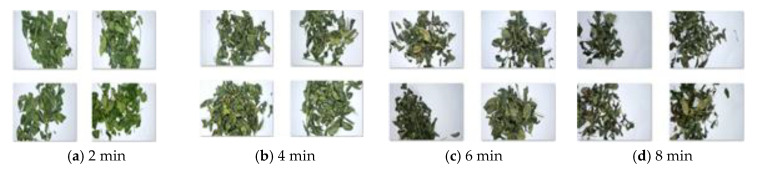
Training samples with different fixation time.

For the difference at the training sample times, batch training is adopted. Each training sample needs to record its corresponding data to facilitate the subsequent analysis and visualization of the results. The training samples collected at different time are shown in Figure 10.

#### 3.2.1. Verification of the Trend and Change of Image Energy

**Figure 11:**
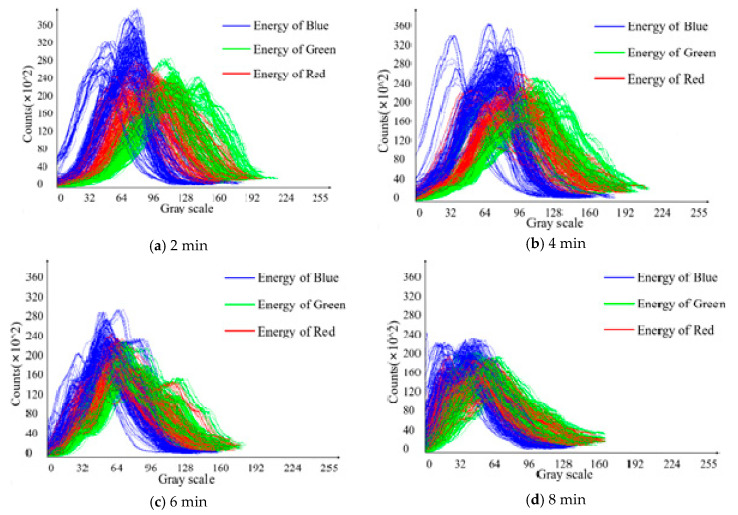
Energy distribution curve of adaptive filtering corresponding to images at different fixation time.

Since the space is limited, the outcome of the background and overlap energy filtering is not added repeatedly, as shown in Figure 4c. The energy distribution curve of adaptive filtering corresponding to images at different fixation time is shown in Figure 11. It has no unit because the corresponding data is dimensionless. A unique global minimum point can be found on the left and right of each curve. These two points are used as threshold points for adaptive background and overlap filtering. It can remove the influence of the background and overlap to the maximum extent, and at the same time, it can retain the energy information of the tea itself in different colors to the corresponding gray scale. 

#### 3.2.2. Verification of the Variation Trend of Energy Clustering Center

**Figure 12:**
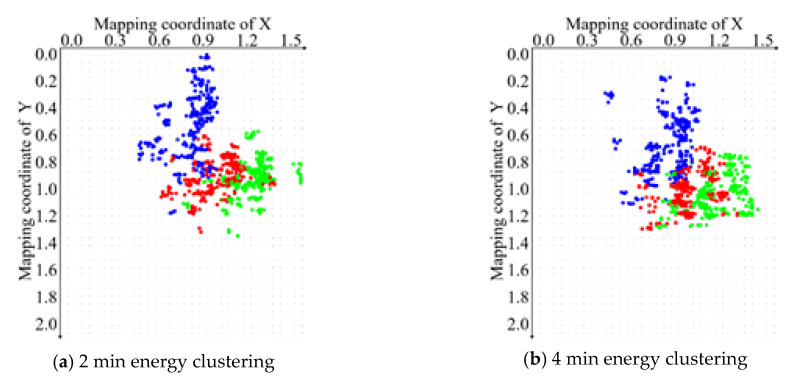

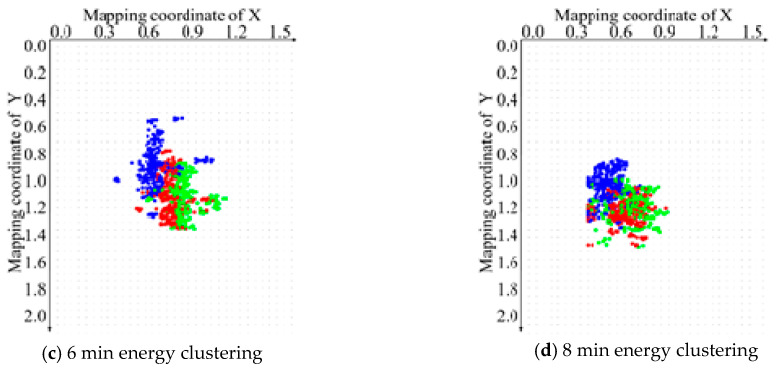
Energy clustering center distributions at different fixation time.

After adaptive filtering, energy data mapping is needed, and the energy data distribution of the corresponding sample at each moment can be determined by feeding into the trainer, as shown in Figure 12.

According to Figure 11 and Figure 12, from the distribution of the energy clustering points of different color channels, the energy of the blue channel varies greatly compared to the different fixation time. The main reason is that the camera is affected by the ambient light and the camera parameters. Therefore, in order to obtain more accurate results, it is better to design multi-angle annular light source illumination by shielding any ambient light.

With the increase of the time interval, the variation trend of energy clustering is nonlinear. This indicates that the related chemical substances in tea also have a similar change and trend. When the fixation time is 2 min, the clustering centers of different color channel energies of different samples are relatively distributed. By 8 min, the clustering centers of different color channel energies are gradually gathered, and the degree of dispersion is significantly decreased. 

This change is in accordance with the actual process of the tea color overall change and trend. In general, with the increase of fixation time, the clustering centers of fixation energy show non-linear aggregation, and the overall change and trend is that it will eventually aggregate in a small, fixed area. The main process we need to do is to control the tea fixation energy gathering in the range of dynamic changes we need, based on the fixation state monitoring.

### 3.3. Euclidean Distance Change and Trend of the Energy Cluster Center of the Three Channels of Training Samples with the Fixation Time Increased

Based on the analysis and statistics of the results obtained by the trainer, the tea image energy clustering training change and trend at different times were generated, and the feature data of each time were recorded in detail in Table 1.

**Figure 13:**
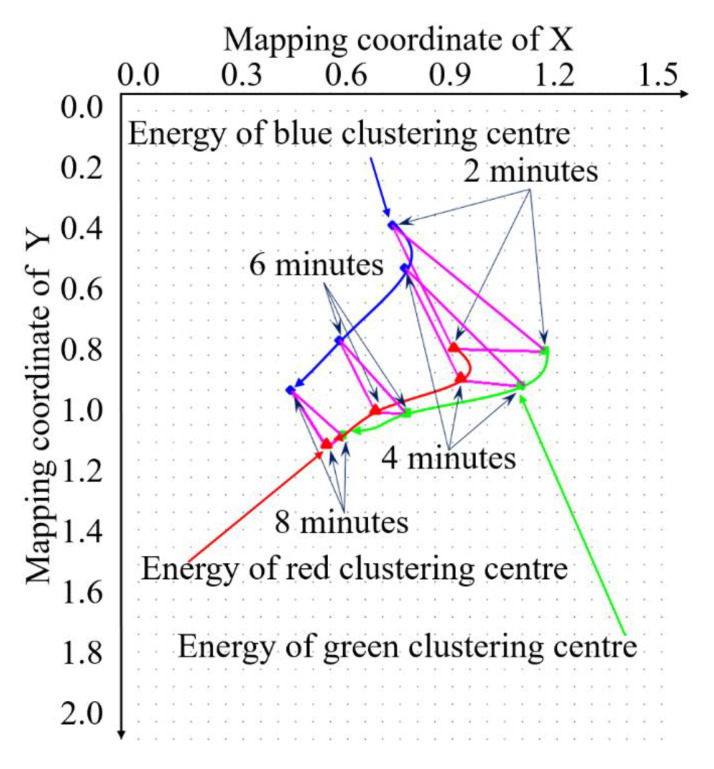
Variation of energy clustering trend of training samples with different fixation time.

According to the distribution data of energy clustering center in different fixation times, the energy clustering curve varying with time can be generated, as shown in Figure 13.

According to the control Equation (21), the Euclidean distance between different color channel centers can be calculated. The detailed data are shown in Table 2. 

**Figure 14:**
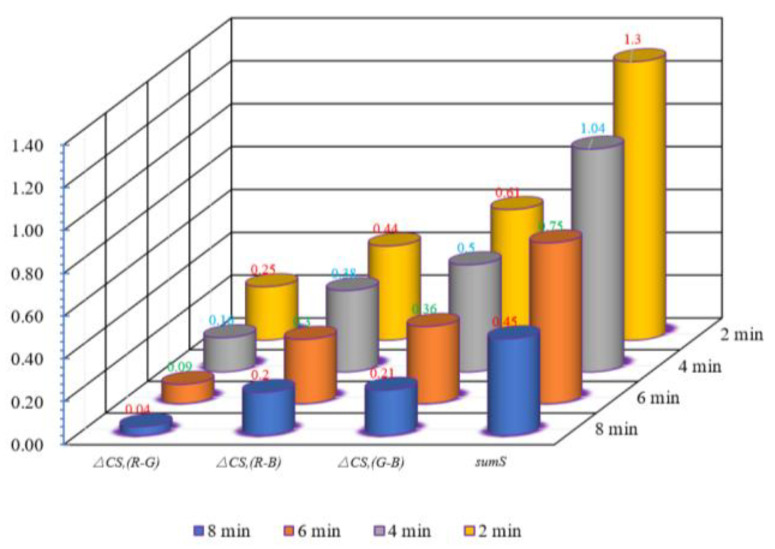
The change and trend of Δ*C_S,(R-G)_*, Δ*C_S,(R-B)_*, Δ*C_S,(G-B)_*, and *sum_S_* with the increase of the fixation time.

It can be observed from Figure 13 and Table 2 that, with the increase in the fixation time, the distance between the energy clustering center of red and blue, red and green, and green and blue get progressively closer. The trend shows a nonlinear relationship—that is, the center distance is not proportional with the same time interval. The data in Table 2 is dimensionless and can be expressed as decimals and integers. As long as the corresponding data are changed in the same proportion, they can be used as the judgment standard. The data intuitively reflects to the changes at Δ*C_S,_*_(*R-G*)_, Δ*C_S,_*_(*R-B*)_, Δ*C_S,_*_(*G-B*)_, and *sum_S_* as the fixation time increases, as shown Figure 14.

### 3.4. Algorithm Comparison Based on Fixation Image

The total number of test samples was 3000, out of which 30 pieces were taken as one set, and a total of 100 sets were taken as test samples. In the experiment, the tea sample corresponding to the training results of 6 min was set to the ideal fixation state. Since the experimental conditions and the method proposed in this paper are mainly based on the image information, it is only compared with the existing analysis-methods-based image. The comparison methods are as follows: gray-level co-occurrence matrix (GLCM); histogram of oriented gridient (HOG) [35]; hue, saturation, value (HSV) + support vector machine (SVM) [36]; light and shade comparison (LASC); and image energy attention mechanism and supervised clustering (IEAMSC) is proposed in this paper. Parameter *α* in the test is 0.2; *H* = 800; *S* = 25,000. The differences in principle between the existing methods and the current method are as follows:

(1) GLCM can reflect the comprehensive information of image gray about direction, adjacent interval, change amplitude, etc. It is the basis for analyzing local patterns and their arrangement rules. GLCM’s disadvantage is that texture features make it difficult to accurately reflect the differences between different textures due to the influence of illumination and reflection. 

(2) HOG is a commonly used method to describe the features of local texture of an image in the fields of computer vision and pattern recognition. The values of gradients in different directions in an area of the image are calculated, and then a histogram is obtained, which can represent the characteristics of the analyzed area. Due to the HOG based gradient, it is sensitive to noise. 

(3) HSV focuses on color representation. Compared with RGB space, HSV space can intuitively express brightness, tone, and the brightness of colors. Therefore, HSV is convenient for color contrast, but it is sensitive to background changes and susceptible to environmental light.

(4) LASC is a direct comparison of the average gray values of three channels of an image. It is easily affected by the background and overlap area, which can easily lead to the fluctuation of the results.

(5) IEAMSC filters out the interference of background and overlap area and realizes the recognition of key features by the attention mechanism. The characteristic clustering ensures the stability under the interference of a single sample.

The complete comparison outcomes are as shown in Table 3. The true positive (TP), true negative (TN), false positive (FP), and false negative (FN) accuracies = (TP + TN)/(TP + FP + TN + FN); precision = TP/(TP + FP); and recall = TP/(TP + FN). T and F are the prediction results true and false, respectively; N and P are the labels of negative and positive respectively; and the precision is the percentage of the total predicted positive samples that are actually positive samples. Recall represents the percentage of positive samples predicted as positive.

## 4. Discussion

By analyzing the data in Table 3, it is clear that the algorithm has achieved a relatively high precision prediction compared with other methods. The GLCM + SVM test results in the wrong parts. This is due to the background information accounting for a large proportion of the features, and the gray value of the local information of tea leaves at different fixation time has a small difference. 

The HOG + SVM test results are such because HOG is based on the gradient feature and the orientation of tea leaves is random, which leads to the error of gradient information. For HSV + SVM, the gray interval needs to be compressed, which will lead to the loss of local information. The background, ambient light, and the tea accounted area of an image have a big impact for the LASC. For the IEAMSC test, the results projects the wrong parts, and this is due to the collected images being greatly influenced by the ambient light, and the data dispersion is more obvious. The tea leaves at 6 min and 8 min are darker in color and relatively less affected by ambient light. Therefore, when conditions permit, it is necessary to shield the ambient light as much as possible and configure a high-brightness multi-angle annular light source.

The fixed focal length and high FPS lens can also ensure the quality of the picture. The selection of the threshold value of the decision function can be made according to the requirements of different tea leaves and different qualities. It also affects accuracy, precision, and recall. Another factor that may affect the test results is that the tea leaves are not uniformly heated in the fixation roller. This will cause color differences at the same fixation time. In summary, the variation trend of training sample energy clustering is consistent. The fixation time increases from the experimental results, indicating that the mechanism of the algorithm is feasible. The test samples showed that a higher detection accuracy rate was realized, which indicates that the algorithm has positive guiding significance in practical applications.

## 5. Conclusions

In order to realize the continuous monitoring of tea fixation status, an online tea fixation processing state monitoring algorithm based on the image energy attention mechanism and supervised clustering (IEAMSC) was proposed. The characteristics and technical contributions of the proposed algorithm are summarized as follows:

(1) An adaptive filtering algorithm is mainly designed for the interference of the background and overlap areas in the image. The filtering threshold can be automatically changed according to the characteristics of each fixation tea image. In this way, the filter is adaptive to each picture, which reduces the filtering of useful tea information by fixed threshold to a certain extent and ensures the accuracy of energy extraction. This indicates that the adaptive filtering is better than the fixed thresholds.

(2) The designed energy extractor is based on the complete energy information of the three channels of the tea image after filtering. The calculation of the energy depends on each individual pixel. The extracted feature data will not have an impact at different angles, and thus, the algorithm has rotation invariance. The energy attention mechanism focuses on the transparent color characteristics of each individual channel of an image, which is similar to the overall color difference evaluation of an image when observed by the human eye. Therefore, the energy attention mechanism can capture the important information of the tea and realizes the dimensionality reduction of data. 

(3) We have found that if there is an increase in the fixation time, and the Euclidean distance of the energy clustering within the center of the three channels of the training samples has gradually approached. These three points formed a triangle that has a trend of gradually shrinking in circumference and area. This change trend is similar to the characteristic change of the tea in actual tea processing when the fixation time increases. We can directly judge the tea status according to the area of this triangle.

(4) The experimental results show that the proposed algorithm has 94% precision compared with other algorithms, and the accuracy has been improved. It indicates that the algorithm proposed in this paper has positive guiding and reference significance for research on the quality control algorithm of the fixation process in an entirely automatic production line.

The points worthy study in the future are to integrate all the feature information in Table 1. The more comprehensive decision functions need to be designed. The result of the fixation does not meet the requirements to not only give the states signal but, at the same time, the accurate fixation time recommendations are also provided. 

## Figures and Tables

**Table 1:** Statistical data of tea image energy clustering training at different fixation times.

Time	*C_S,R_(x)*	*C_S,R_(y)*	*C_S,G_(x)*	*C_S,G_(y)*	*C_S,B_(x)*	*C_S,B_(y)*	*h(R)*	*h(G)*	*h(B)*	*f (R)*	*f (G)*	*f (B)*
**2 min**	0.91	0.80	1.16	0.82	0.72	0.40	70	102	72	271	275	380
**4 min**	0.93	0.88	1.09	0.91	0.76	0.53	95	107	65	254	246	352
**6 min**	0.68	1.02	0.77	1.03	0.56	0.74	62	66	64	241	232	281
**8 min**	0.54	1.12	0.58	1.10	0.43	0.95	56	57	56	182	195	221

**Table 2:** Calculation and comparison of clustering data at different fixation times.

Time	Δ*C_S,(R-G)_*	Δ*C_S,(R-B)_*	Δ*C_S,(G-B)_*	*Sum_S_*
**2 min**	0.25	0.44	0.61	1.30
**4 min**	0.16	0.38	0.50	1.04
**6 min**	0.09	0.30	0.36	0.75
**8 min**	0.04	0.20	0.21	0.45

**Table 3:** Comparison of detection results of different methods based on image information.

Name	GLCM + SVM	HOG + SVM	HSV + SVM	LASC	IEAMSC
**Accuracy**	74%	61%	66%	54%	95%
**Precision**	72%	60%	64%	50%	94%
**Recall**	75%	61%	67%	54%	96%
